# Suicidal Ideation Among College Students: cross-sectional study

**DOI:** 10.1192/j.eurpsy.2025.2362

**Published:** 2025-08-26

**Authors:** Z. Nesrine, I. Gassara, N. Smaoui, R. Feki, N. Charfi, J. Ben Thabet, L. Zouari, M. Maalej, M. Maalej, S. Omri

**Affiliations:** 1 pédopsychitrie; 2 psychiatrie C, hopital hedi chaker sfax, sfax, Tunisia

## Abstract

**Introduction:**

Suicidal ideation, encompassing thoughts, planning, and the desire for death, is a critical facet of the suicidal process, potentially leading to suicide attempts and completion. Understanding its prevalence and severity among college students is vital for preventive interventions.

**Objectives:**

Our study aimed to estimate the prevalence and severity of suicidal ideation among college students.

**Methods:**

We conducted a cross-sectional and analytical study among students from various faculties in Sfax between October 2022 and January 2023. Student recruitment occurred electronically through a Google Forms questionnaire, emphasizing anonymity and the study’s scientific purpose. We utilized the Suicidal Ideation Attributes Scale (French Version) (SIDAS-FR) to identify and assess the severity of suicidal ideation.

**Results:**

Our sample comprised 149 students, predominantly female (83,20%), with an average age of 26 years. Among them, 78,5% were single, and 81,9% lived with their families. Nearly half of the students were from the Sfax Faculty of Medicine, and 64,4% were in their 3rd cycle of education. The mean total score on the SIDAS scale was 1,21 +/- 3,84. Suicidal ideation was reported by 11,4% of participants, with 5,36% indicating moderate to high severity. Factors associated with suicidal ideation included psychiatric disorders (p=0,00), alcohol consumption (p=0,033), psychotropic medication use (p=0,001), and unsatisfactory intrafamily communication (p=0,036).

**Conclusions:**

Suicidal ideation, a concerning issue, particularly among young people, demands focused attention in public health efforts. Understanding the associated factors is pivotal for prevention strategies, emphasizing the importance of mental health support and effective communication within families.

**Disclosure of Interest:**

None Declared

